# Functional and oncological outcomes after laparoscopic versus open total mesorectal excision for rectal cancer: a propensity score-matched study

**DOI:** 10.3389/fsurg.2026.1961595

**Published:** 2026-10-20

**Authors:** Xuetong Jiang, Chuang Ding, Ming Zhang, Yan Chen, Lingyong Kong

**Affiliations:** 1 Department of Gastrointestinal Surgery, The Affiliated Suqian Hospital of Xuzhou Medical University, Suqian, China; 2 Department of Gastrointestinal Surgery, Nanjing Drum Tower Hospital Group Suqian Hospital, Suqian, Jiangsu, China

**Keywords:** anal function, laparoscopic surgery, LARS, open surgery approach, rectal cancer

## Abstract

**Background:**

While minimally invasive surgery and traditional open surgery are generally equivalent in terms of oncological outcomes for rectal cancer, maintaining postoperative quality of life is increasingly important. Low anterior resection syndrome is a distressing complication following sphincter-preserving surgery that severely impacts a patient's normal life. This study aims to systematically compare the short-term clinical efficacies of laparoscopic total mesorectal excision and open radical surgery, using LARS scoring system to objectively evaluate postoperative anal function.

**Methods:**

A retrospective, single-center analysis was conducted on clinical data from patients who underwent radical resection for mid-low rectal cancer at Nanjing Drum Tower Hospital Group Suqian Hospital from January 2020 to March 2023. Following strict inclusion and exclusion criteria, 361 patients were initially selected. To mitigate baseline imbalances, a 1:2 propensity score matching was utilized, yielding a final matched cohort of 160 patients in the LaTME (laparoscopic total mesorectal excision) group and 80 patients in the OsTME (open total mesorectal excision) group.

**Results:**

Both groups demonstrated highly comparable oncological efficacy, achieving negative circumferential resection margins in 97.50% of cases and complete total mesorectal excision in over 96.00% of cases. Additionally, 1-year and 3-year disease-free survival and overall survival profiles showed no statistically significant differences between the two modalities. While systemic postoperative complications were similar between groups, the LaTME cohort experienced a significantly lower rate of postoperative urinary retention (1.90%) compared to the OsTME cohort (8.80%, *p* = 0.030). For patients without a diverting ileostomy, the LaTME group had a notably lower incidence of severe LARS at one month postoperatively (23.35%) compared to the OsTME group (35.93%, *p* = 0.009). This functional disparity diminished by six months, at which point both groups equalized. Subgroups requiring an ileostomy showed no significant differences in LARS scores following stoma reversal.

**Conclusion:**

Laparoscopic total mesorectal excision provides equivalent oncological outcomes to traditional open surgery for mid-low rectal cancer. LaTME offers superior short-term functional benefits by reducing postoperative urinary retention and substantially decreasing the severity of early Low Anterior Resection Syndrome. These minimally invasive advantages support prioritizing LaTME to maximize functional preservation and patient quality of life.

## Introduction

Colorectal cancer is one of the most prevalent malignancies globally, with its incidence demonstrating a continuously increasing trend in recent years (1). Although remarkable advancements have been achieved in comprehensive treatment modalities, including neoadjuvant chemoradiotherapy, targeted therapy, and immunotherapy, radical surgery adhering to the principle of total mesorectal excision (TME) remains the sole and fundamental curative strategy for rectal cancer (2). Nevertheless, the specific anatomical location of the rectum and the confined pelvic space make the surgical operation exceedingly complex, thereby presenting severe challenges to surgeons. Consequently, maximizing the reduction of surgical trauma and accelerating postoperative recovery, without compromising oncological radicality, has consistently been a primary focus in colorectal surgery.

With the continuous development of minimally invasive surgery (MIS) techniques, laparoscopic surgery (LaTME) has gradually become the mainstream surgical approach for mid-low rectal cancer due to its inherent advantages such as minimal trauma, mild postoperative pain, shorter hospital stay, and rapid postoperative recovery (3). Several landmark multicenter randomized controlled trials such as the COLOR II trial have confirmed that minimally invasive surgery is equivalent to traditional open surgery (OsTME) in terms of short- and long-term oncological outcomes including disease-free survival and overall survival, demonstrating comparability in the efficacy of oncological radicality (4, 5). However, as the survival time of rectal cancer patients continues to prolong, postoperative quality of life has increasingly drawn the attention of clinicians and patients. Among these, low anterior resection syndrome (LARS) is the most common and extremely distressing complication following sphincter-preserving surgery for rectal cancer, mainly manifesting as defecation dysfunction, which severely impacts the normal life of patients (6). Currently, high-quality clinical studies regarding the exact comparison of anal function preservation between laparoscopic surgery and open surgery for rectal cancer remain controversial or lack sufficient data. The LARS score, as an internationally recognized and validated questionnaire, has been widely applied to scientifically evaluate the severity of postoperative bowel dysfunction (7). Nevertheless, in previous studies comparing these two surgical modalities, literature that specifically utilizes the LARS score for systematic quantitative evaluation of postoperative anal function and employs rigorous statistical methods to control for confounding bias is relatively limited.

Consequently, by conducting a retrospective analysis of a large patient cohort, this study endeavors to systematically compare the short-term clinical efficacies of laparoscopic TME (LaTME) and traditional open radical surgery for rectal cancer, placing a specific emphasis on surgical procedural details and utilizing propensity score matching (PSM). The primary objective is to investigate the potential advantages of the laparoscopic approach in preserving postoperative anal function. Notably, by incorporating the LARS scoring system for an objective assessment, this study aims to validate the superiority of laparoscopic surgery in facilitating the recovery of defecation function. Ultimately, our goal is to provide robust, evidence-based support for surgeons when tailoring personalized surgical strategies for patients with rectal cancer.

## Methods

### Data collection and patient selection

We collected detailed clinical data of patients who underwent radical resection for rectal cancer at Nanjing Drum Tower Hospital Group Suqian Hospital from January 2020 to March 2023. The inclusion criteria were as follows: pathologically confirmed adenocarcinoma by preoperative colonoscopy; distance of the lower tumor margin from the anal verge <15 cm; received open or laparoscopic radical resection adhering to the principle of total mesorectal excision (Dixon); achieved R0 resection for curative intent; no synchronous or metachronous distant metastasis. The exclusion criteria were as follows: failure to achieve radical surgical intent; having undergone Miles' surgery due to tumor invasion of the external anal sphincter; having undergone Hartmann's surgery due to cancerous obstruction; evaluated as stage IV according to the American Society of Anesthesiologists (ASA) score indicating high anesthesia risk; poor preoperative anal function (It is mainly judged based on objective specialist examination criteria. For example, digital rectal examination indicates that the anal resting pressure and maximum squeeze pressure are far below the normal reference values, and the anal sphincter tone is severely weakened or even completely flaccid. Alternatively, anorectal manometry confirms that the anal sphincter pressure has decreased by more than 50% compared with the healthy reference value, suggesting obvious organic impairment of the sphincter function). Postoperative urinary retention was defined as the inability to voluntarily void urine requiring catheterization for more than 48 h postoperatively, or the need for re-catheterization after initial removal, confirmed by a bladder ultrasound residual volume of >300 mL. After strict case screening based on the above inclusion and exclusion criteria, a total of 361 patients were included in this cohort study. After being fully informed of the respective advantages of laparoscopic and open surgery, 88 patients underwent traditional open surgery, while 273 patients underwent laparoscopic surgery (Figure 1). Following a 1:2 propensity score matching analysis, 160 cases were successfully included in the laparoscopic group, alongside 80 cases in the open group. This study was approved by the Ethics Committee of Nanjing Drum Tower Hospital Group Suqian Hospital (2026-017-01) and complied with the 1964 Helsinki Declaration.

**Figure 1:**
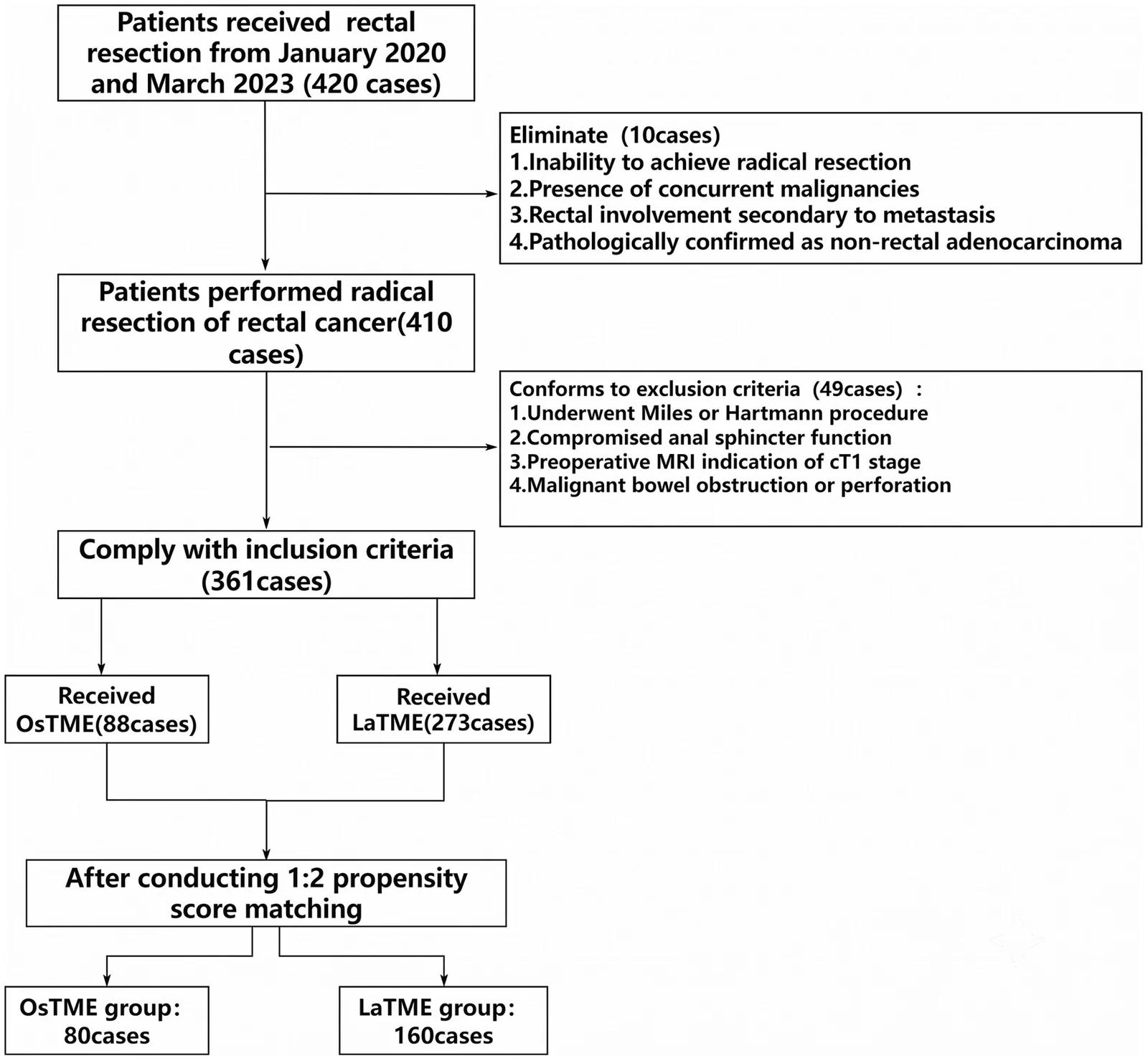
Flow diagram of patient selection.

### Patient management and clinical workflow

All patients were admitted via the outpatient clinic presenting with various symptoms. Following a digital rectal examination (DRE) and other physical evaluations by the primary physician, patients underwent colonoscopy with multi-site endoscopic biopsies for subsequent pathological diagnosis, which confirmed rectal adenocarcinoma. To determine local tumor staging, pelvic magnetic resonance imaging (MRI) was performed to assess the depth of tumor invasion and potential involvement of the surrounding sphincters. Additionally, high-resolution chest and abdominal computed tomography (CT) scans were conducted to evaluate for distant metastasis. Furthermore, auxiliary pulmonary and cardiac function assessments were performed to minimize anesthesia-related risks.

In accordance with clinical protocols, patients with cT3-T4 or N+ disease were recommended to receive three cycles of fluorouracil- and platinum-based preoperative neoadjuvant chemotherapy; radiotherapy was not recommended. After being fully informed about the characteristics of the two surgical modalities, the patients independently selected their surgical approach based on their comprehensive personal circumstances, including financial considerations. Regarding the creation of a defunctioning ileostomy, individualized risk scoring was conducted using a predictive model established from large-sample clinical studies. A defunctioning ileostomy was performed for high-risk patients (Figure 2) (8). Postoperatively, these patients were instructed to perform pelvic floor muscle training, and stoma reversal surgery was scheduled six months after the initial operation.

**Figure 2:**
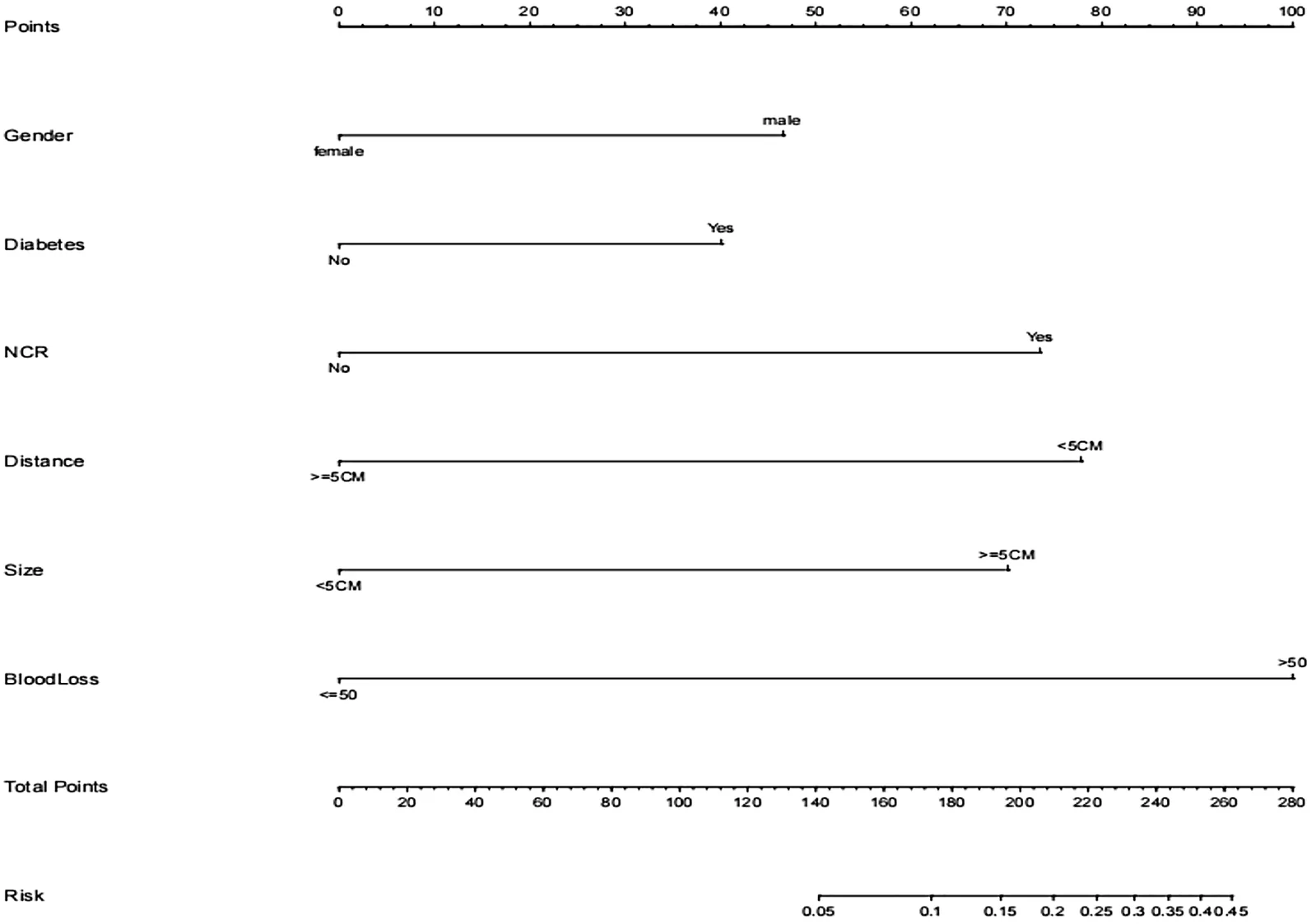
Nomogram prediction model of anastomotic leakage.

### Surgical technique and detail

The procedures of the two surgical approaches are generally similar, and both are guided by the “Chinese Expert Consensus on Robotic Surgery for Colorectal Cancer (2020 Edition)”. The surgery is performed via a medial approach, entering the sigmoid mesocolon through the projection of the sacral promontory. The No. 253 lymph nodes are dissected at the root of the inferior mesenteric artery, and the left colic artery is carefully identified and preserved. During this process, the space of Denonvilliers' fascia is entered, with precise protection of the ureters and autonomic nerve plexuses. Subsequently, the dissection proceeds into the space between the fascia propria of the rectum and the presacral fascia, mobilizing the rectum bilaterally from the posterior to the anterior. After complete transection of the rectum, anastomosis of the remaining bowel is performed, typically using a 29-mm circular stapler. Following the stapled anastomosis, reinforcing sutures are placed using absorbable sutures, and an air leak test is conducted to verify the integrity of the anastomosis (Figure 3).

**Figure 3:**
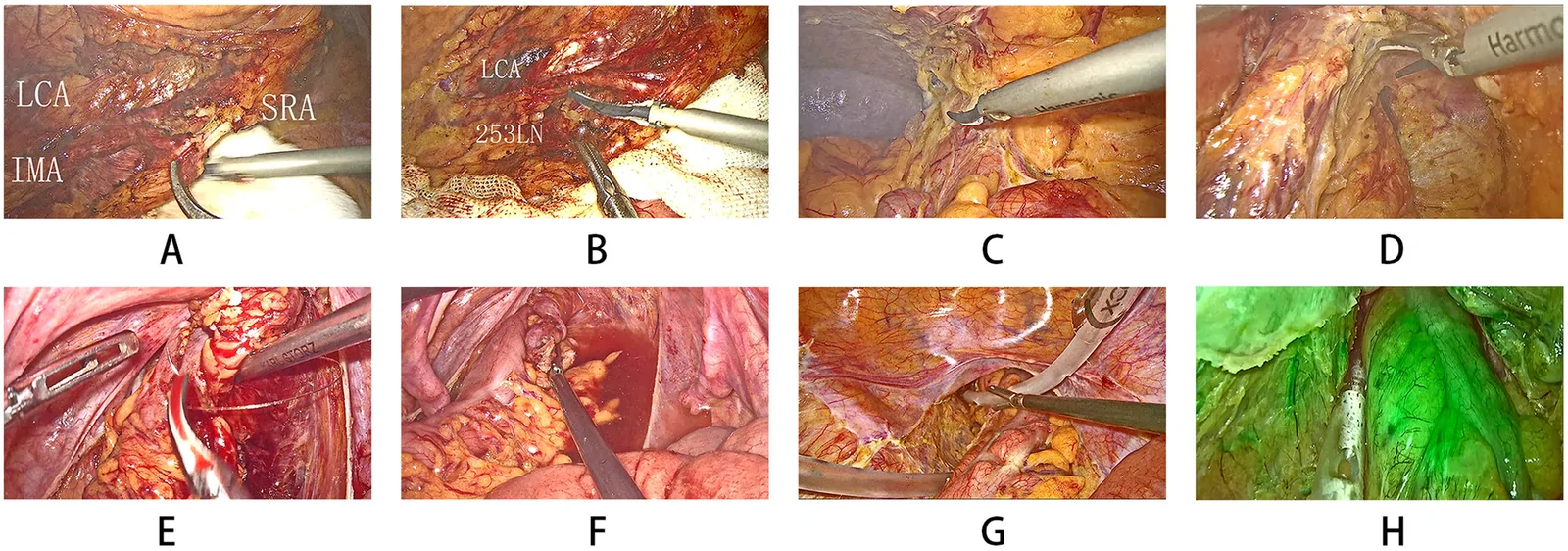
**(A)** Preservation of the left colic artery. **(B)** Dissected No. 253 lymph nodes. **(C)** Mobilization of the splenic flexure. **(D)** Separated the mesorectum and Denonvilliers’ fascia principled by TME. **(E)** Reinforcing suture of the anastomosis. **(F)** Air leak test. **(G)** Placement of a drainage tube. **(H)** Indocyanine green (ICG) fluorescence navigation.

### Primary objectives and observation indicators

The primary objective of this study is to compare the advantages and disadvantages of the two surgical approaches in terms of anal function from the perspective of surgical details. Although there are various methods to evaluate anal function, such as objective assessment using instruments like anorectal manometry, it is difficult to obtain complete data due to patient compliance issues. Therefore, the LARS scoring scale was selected for statistical analysis. The secondary objective is to compare the two surgical methods in terms of oncological outcomes, such as disease-free survival (DFS) and overall survival (OS), as well as the incidence of postoperative complications.

### Statistical analysis

All statistical analyses were performed using SPSS version 26.0. To mitigate baseline imbalances between the two groups, a 1:2 propensity score matching (PSM) was conducted utilizing the nearest-neighbor method with a caliper width of 0.02, incorporating baseline characteristics as covariates. Categorical variables are presented as frequencies and percentages (*n*, %) and were compared using the Chi-square test. Overall survival and disease-free survival were estimated using the Kaplan–Meier method and compared via the log-rank test, which was executed using the “survival” package in RStudio. A two-sided *p* < 0.05 was considered statistically significant.

## Results

### Baseline characteristics before and after propensity score matching

Before propensity score matching, the study cohort comprised 273 patients who underwent LaTME and 88 patients who underwent OsTME. The baseline clinicopathological characteristics were largely comparable between the two groups, except for the clinical N category (*p* = 0.0042) and pathological T category (*p* = 0.021), which demonstrated statistically significant differences. To minimize selection bias and balance the baseline variables, propensity score matching was applied, yielding a matched cohort of 160 patients in the LaTME group and 80 patients in the OsTME group. Following this matching process, all baseline demographic, clinical, and operative variables were successfully balanced with no significant differences observed (all *p* > 0.05). These balanced variables included age, gender, body mass index, smoking and alcohol history, as well as comorbidities such as diabetes, hypertension, ischemic heart disease, and chronic obstructive pulmonary disease. Furthermore, tumor-related characteristics and operative details—including clinical and pathological T and N categories, tumor size, distance from the anal verge, receipt of neoadjuvant chemoradiotherapy, specimen length, operative time, estimated blood loss, and the creation of a diverting stoma—were also well-matched between the two cohorts (Table 1).

**Table 1:** Demographic and perioperative characteristics.

	Before propensity score matching				After propensity score matching			
	LaTME (*N* = 273)	OsTME (*N* = 88)	*χ* ^2^	*P*-value	LaTME (*N* = 160)	OsTME (*N* = 80)	*χ* ^2^	*P*-value
Age			0.651	0.42			0.354	0.552
<65	152 (55.7%)	44 (50.0%)			88 (55.0%)	40 (50.0%)		
=>65	121 (44.3%)	44 (50.0%)			72 (45.0%)	40 (50.0%)		
Gender			0.494	0.482			0.353	0.552
Female	132 (48.4%)	47 (53.4%)			82 (51.3%)	45 (56.3%)		
Male	141 (51.6%)	41 (46.6%)			78 (48.8%)	35 (43.8%)		
BMI			0.461	0.497			2.097	0.148
<24 kg/m^2^	212 (77.7%)	72 (81.8%)			117 (73.1%)	66 (82.5%)		
=>24 kg/m^2^	61 (22.3%)	16 (18.2%)			43 (26.9%)	14 (17.5%)		
Smoking			0.785	0.376			1.820	0.177
No	209 (76.6%)	72 (81.8%)			118 (73.8%)	66 (82.5%)		
Yes	64 (23.4%)	16 (18.2%)			42 (26.3%)	14 (17.5%)		
Alcohol			0.007	0.934			0.000	1
No	161 (59.0%)	53 (60.2%)			95 (59.4%)	47 (58.8%)		
Yes	112 (41.0%)	35 (39.8%)			65 (40.6%)	33 (41.3%)		
Diabetes			0.218	0.64			2.390	0.122
No	215 (78.8%)	72 (81.8%)			116 (72.5%)	66 (82.5%)		
Yes	58 (21.2%)	16 (18.2%)			44 (27.5%)	14 (17.5%)		
Hypertension			0.000	1			0.003	0.953
No	223 (81.7%)	72 (81.8%)			130 (81.3%)	66 (82.5%)		
Yes	50 (18.3%)	16 (18.2%)			30 (18.8%)	14 (17.5%)		
Ischemic.heart.disease			3.771	0.0521			2.467	0.116
No	264 (96.7%)	80 (90.9%)			155 (96.9%)	73 (91.3%)		
Yes	9 (3.3%)	8 (9.1%)			5 (3.1%)	7 (8.8%)		
COPD			0.000	1			0.000	1
No	222 (81.3%)	72 (81.8%)			133 (83.1%)	66 (82.5%)		
Yes	51 (18.7%)	16 (18.2%)			27 (16.9%)	14 (17.5%)		
Clinical.T.category			0.513	0.474			0.385	0.535
T1–2	92 (33.7%)	34 (38.6%)			54 (33.8%)	31 (38.8%)		
T3–4	181 (66.3%)	54 (61.4%)			106 (66.3%)	49 (61.3%)		
Clinical.N.category			8.193	0.0042			8.087	0.0657
N0	177 (64.8%)	72 (81.8%)			100 (62.5%)	66 (82.5%)		
N1–2	96 (35.2%)	16 (18.2%)			60 (37.5%)	14 (17.5%)		
nCRT			0.218	0.64			2.390	0.122
No	58 (21.2%)	16 (18.2%)			44 (27.5%)	14 (17.5%)		
Yes	215 (78.8%)	72 (81.8%)			116 (72.5%)	66 (82.5%)		
Tumour.sizs			0.000	1			0.111	0.739
<=5 cm	178 (65.2%)	57 (64.8%)			101 (63.1%)	53 (66.3%)		
>5 cm	95 (34.8%)	31 (35.2%)			59 (36.9%)	27 (33.8%)		
Distance.from.AV			0.561	0.454			2.097	0.148
<=5 cm	62 (22.7%)	16 (18.2%)			43 (26.9%)	14 (17.5%)		
>5 cm	211 (77.3%)	72 (81.8%)			117 (73.1%)	66 (82.5%)		
Length.of.specimen			0.042	0.837			0.196	0.658
<=15 cm	45 (16.5%)	16 (18.2%)			23 (14.4%)	14 (17.5%)		
>15 cm	228 (83.5%)	72 (81.8%)			137 (85.6%)	66 (82.5%)		
Operative.time			0.611	0.434			0.103	0.748
<=180 min	133 (48.7%)	38 (43.2%)			73 (45.6%)	34 (42.5%)		
>180 min	140 (51.3%)	50 (56.8%)			87 (54.4%)	46 (57.5%)		
Blood.loss			0.371	0.542			0.887	0.346
<=20 mL	213 (78.0%)	72 (81.8%)			122 (76.3%)	66 (82.5%)		
>20 mL	60 (22.0%)	16 (18.2%)			38 (23.8%)	14 (17.5%)		
Diverting.stoma			0.279	0.597			0.174	0.677
No	226 (82.8%)	70 (79.5%)			133 (83.1%)	64 (80.0%)		
Yes	47 (17.2%)	18 (20.5%)			27 (16.9%)	16 (20.0%)		
Pathological.T.category			5.323	0.021			3.380	0.066
T1–2	91 (33.3%)	42 (47.7%)			53 (33.1%)	37 (46.3%)		
T3–4	182 (66.7%)	46 (52.3%)			107 (66.9%)	43 (53.8%)		
Pathological.N.category			0.009	0.926			0.052	0.819
N0	136 (49.8%)	45 (51.1%)			82 (51.3%)	43 (53.8%)		
N1–2	137 (50.2%)	43 (48.9%)			78 (48.8%)	37 (46.3%)		

### Surgical outcomes and postoperative complications

Regarding the oncological efficacy and quality of the surgical resection, an analysis of the propensity score-matched cohorts demonstrated comparable outcomes between the LaTME and OsTME groups. Specifically, negative circumferential resection margins (CRM) were successfully achieved in the vast majority of cases, accounting for 156 of 160 patients (97.50%) in the LaTME group and 78 of 80 patients (97.50%) in the OsTME group (*p* = 1). Correspondingly, positive CRM was rarely observed, occurring in only 4 patients (2.50%) and 2 patients (2.50%) in the LaTME and OsTME cohorts, respectively. Furthermore, macroscopic assessment of the surgical specimens revealed that a complete total mesorectal excision (TME) was accomplished in 157 patients (98.10%) undergoing LaTME and 77 patients (96.30%) undergoing OsTME. A nearly complete TME was noted in a minimal fraction of the matched cohorts, specifically 3 patients (1.90%) in the LaTME group and 3 patients (3.80%) in the OsTME group, yielding no statistically significant difference in the overall quality of TME between the two surgical approaches (*p* = 0.661). When evaluating intraoperative safety, the incidence of adverse surgical events was found to be exceedingly low and homogeneously distributed across both treatment arms. Presacral bleeding was documented in only 3 patients (1.90%) within the LaTME group and 2 patients (2.50%) in the OsTME group (*p* = 1). Similarly, ureteric injury occurred in 3 cases (1.90%) during LaTME compared to 2 cases (2.50%) during OsTME (*p* = 1). The rate of iatrogenic rectal perforation was also minimal, affecting 4 patients (2.50%) managed with LaTME and 2 patients (2.50%) managed with OsTME (*p* = 1).

In terms of the postoperative recovery profile, the occurrence of short-term complications was generally comparable across both surgical modalities after propensity score matching, with most specific complications showing no statistically significant disparities. Anastomotic leakage, a critical postoperative concern, was detected in 13 patients (8.10%) in the LaTME group and 4 patients (5.00%) in the OsTME group (*p* = 0.533). Other severe intra-abdominal complications also presented at similar, low frequencies, including pelvic abscesses and bleeding in the peritoneal cavity for the LaTME and OsTME groups, respectively. Systemic postoperative complications similarly showed no significant variance; pulmonary infections were recorded in 27 patients (16.90%) following LaTME and 14 patients (17.50%) following OsTME, while deep vein thrombosis was exceptionally rare, occurring in just 1 patient (0.60%) and 1 patient (1.30%) in the respective groups (*p* = 1). However, a notable and statistically significant exception was observed in the genitourinary functional outcomes. The incidence of postoperative urinary retention was markedly lower in patients who underwent LaTME compared to those who received OsTME. Specifically, only 3 patients (1.90%) in the LaTME cohort experienced urinary retention, whereas 7 patients (8.80%) in the OsTME cohort developed this complication, yielding a statistically significant advantage for the laparoscopic approach (*p* = 0.030) (Table 2).

**Table 2:** Pathological characteristics and clinical outcomes.

	Before propensity score matching				After propensity score matching			
	LaTME (*N* = 273)	OsTME (*N* = 88)	*χ* ^2^	*P*-value	LaTME (*N* = 160)	OsTME (*N* = 80)	*χ* ^2^	*P*-value
CRM			0.058	0.81			0.000	1
Negative	267 (97.8%)	85 (96.6%)			156 (97.5%)	78 (97.5%)		
Positive	6 (2.2%)	3 (3.4%)			4 (2.5%)	2 (2.5%)		
Quality.of.TME			0.630	0.427			0.192	0.661
Complete	267 (97.8%)	84 (95.5%)			157 (98.1%)	77 (96.3%)		
Nearly complete	6 (2.2%)	4 (4.5%)			3 (1.9%)	3 (3.8%)		
Presacral.bleeding			0.498	0.48			0.000	1
No	269 (98.5%)	85 (96.6%)			157 (98.1%)	78 (97.5%)		
Yes	4 (1.5%)	3 (3.4%)			3 (1.9%)	2 (2.5%)		
Ureteric.injury			0.210	0.647			0.000	1
No	268 (98.2%)	85 (96.6%)			157 (98.1%)	78 (97.5%)		
Yes	5 (1.8%)	3 (3.4%)			3 (1.9%)	2 (2.5%)		
Rectal.perforation			0.210	0.647			0.000	1
No	268 (98.2%)	85 (96.6%)			156 (97.5%)	78 (97.5%)		
Yes	5 (1.8%)	3 (3.4%)			4 (2.5%)	2 (2.5%)		
Anastomotic.leak			0.369	0.544			0.388	0.533
No	250 (91.6%)	83 (94.3%)			147 (91.9%)	76 (95.0%)		
Yes	23 (8.4%)	5 (5.7%)			13 (8.1%)	4 (5.0%)		
Pelvic.abscess			0.000	1			0.000	1
No	271 (99.3%)	87 (98.9%)			158 (98.8%)	79 (98.8%)		
Yes	2 (0.7%)	1 (1.1%)			2 (1.3%)	1 (1.3%)		
Bleeding.in.peritoneal.cavity			0.000	1			0.000	1
No	271 (99.3%)	87 (98.9%)			159 (99.4%)	79 (98.8%)		
Yes	2 (0.7%)	1 (1.1%)			1 (0.6%)	1 (1.3%)		
Urinary.retention			3.771	0.0521			4.709	0.03
No	264 (96.7%)	80 (90.9%)			157 (98.1%)	73 (91.3%)		
Yes	9 (3.3%)	8 (9.1%)			3 (1.9%)	7 (8.8%)		
Pulmonary.infection			0.000	1			0.000	1
No	222 (81.3%)	72 (81.8%)			133 (83.1%)	66 (82.5%)		
Yes	51 (18.7%)	16 (18.2%)			27 (16.9%)	14 (17.5%)		
Deep.vein.thrombosis			0.087	0.768			0.000	1
No	270 (98.9%)	86 (97.7%)			159 (99.4%)	79 (98.8%)		
Yes	3 (1.1%)	2 (2.3%)			1 (0.6%)	1 (1.3%)		

#### Postoperative anal function and long-term oncological outcomes

Postoperative anal function was systematically evaluated utilizing the Low Anterior Resection Syndrome (LARS) score, with patients stratified based on whether they received a diverting ileostomy. Among patients who underwent low anterior resection (LAR) without an ileostomy (133 in the LaTME group and 64 in the OsTME group), a statistically significant difference in bowel function was observed at one month postoperatively (*p* = 0.009). Specifically, while the rates of normal function were comparable (8.12% vs. 9.38%), the LaTME cohort experienced a notably lower incidence of severe LARS (23.35%) compared to the OsTME cohort (35.93%), with the majority of LaTME patients reporting only mild LARS (68.53% vs. 54.69%). However, this functional disparity diminished over time; by six months postoperatively, functional outcomes had significantly improved and equalized between the two surgical arms (*p* = 0.660). At this six-month follow-up, the incidence of severe LARS had decreased to 9.77% in the LaTME group and 14.06% in the OsTME group, while normal bowel function was achieved in 31.58% and 31.25% of patients, respectively. For the subgroup of patients who required an ileostomy and subsequently underwent reversal (27 in the LaTME group and 16 in the OsTME group), no statistically significant differences in LARS scores were detected between the two approaches at either one month (*p* = 0.851) or six months (*p* = 0.944) following stoma closure. By the six-month mark post-reversal, both groups demonstrated comparable functional recovery profiles, with approximately one-third of patients in each cohort achieving normal bowel function (33.33% vs. 31.25%) and severe LARS dropping to 14.81% and 18.75%, respectively. Regarding long-term oncological efficacy, the propensity score-matched analysis revealed comparable survival profiles between the two surgical modalities. Although patients in the LaTME group exhibited marginally higher survival percentages across all temporal milestones, these differences did not reach statistical significance. The 1-year and 3-year disease-free survival (DFS) rates were 95.63% and 91.88% for the LaTME group, compared to 90.00% and 83.75% for the OsTME group (*p* = 0.09 and *p* = 0.060, respectively). Similarly, the overall survival (OS) outcomes were statistically equivalent between the cohorts, demonstrating a 1-year OS of 96.88% vs. 93.75% (*p* = 0.250) and a 3-year OS of 94.38% vs. 88.75% (*p* = 0.120) for the LaTME and OsTME groups, respectively (Table 3) (Figure 4).

**Table 3:** Oncological and functional outcomes.

	After propensity score matching			
	LaTME (*N* = 160)	OsTME (*N* = 80)	*χ* ^2^	*P*-value
1 month after LAR without ileostomy	133	64	*χ*^2^ = 9.27	*P* = 0.009
Normal	10, 8.12%	6, 9.38%		
Mild LARS	100, 68.53%	35, 54.69%		
Severe LARS	23, 23.35%	23, 35.93%		
6 month after LAR without ileostomy			*χ*^2^ = 0.831	*P* = 0.660
Normal	42, 31.58%	20, 31.25%		
Mild LARS	78, 58.65%	35, 54.69%		
Severe LARS	13, 9.77%	9, 14.06%		
1 month after ileostomy				
reversal with ileostomy	27	16	*χ*^2^ = 0.324	*P* = 0.851
Normal	3, 11.11%	2, 11.76%		
Mild LARS	15, 55.56%	8, 47.06%		
Severe LARS	9, 33.33%	7, 41.18%		
6 month after ileostomy				
Reversal			*χ*^2^ = 0.116	*P* = 0.944
Normal	9, 33.33%	5, 31.25%		
Mild LARS	14, 51.85%	8, 50.00%		
Severe LARS	4, 14.81%	3, 18.75%		
1-Year disease-free survival	153, 95.63%	72, 90.00%	*χ*^2^ = 2.88	*P* = 0.09
3-Year disease-free survival	147, 91.88%	67, 83.75%	*χ*^2^ = 3.64	*P* = 0.06
1-Year overall survival	155, 96.88%	75, 93.75%	*χ*^2^ = 1.30	*P* = 0.25
3-Year overall survival	151, 94.38%	71, 88.75%	*χ*^2^ = 2.43	*P* = 0.12

**Figure 4:**
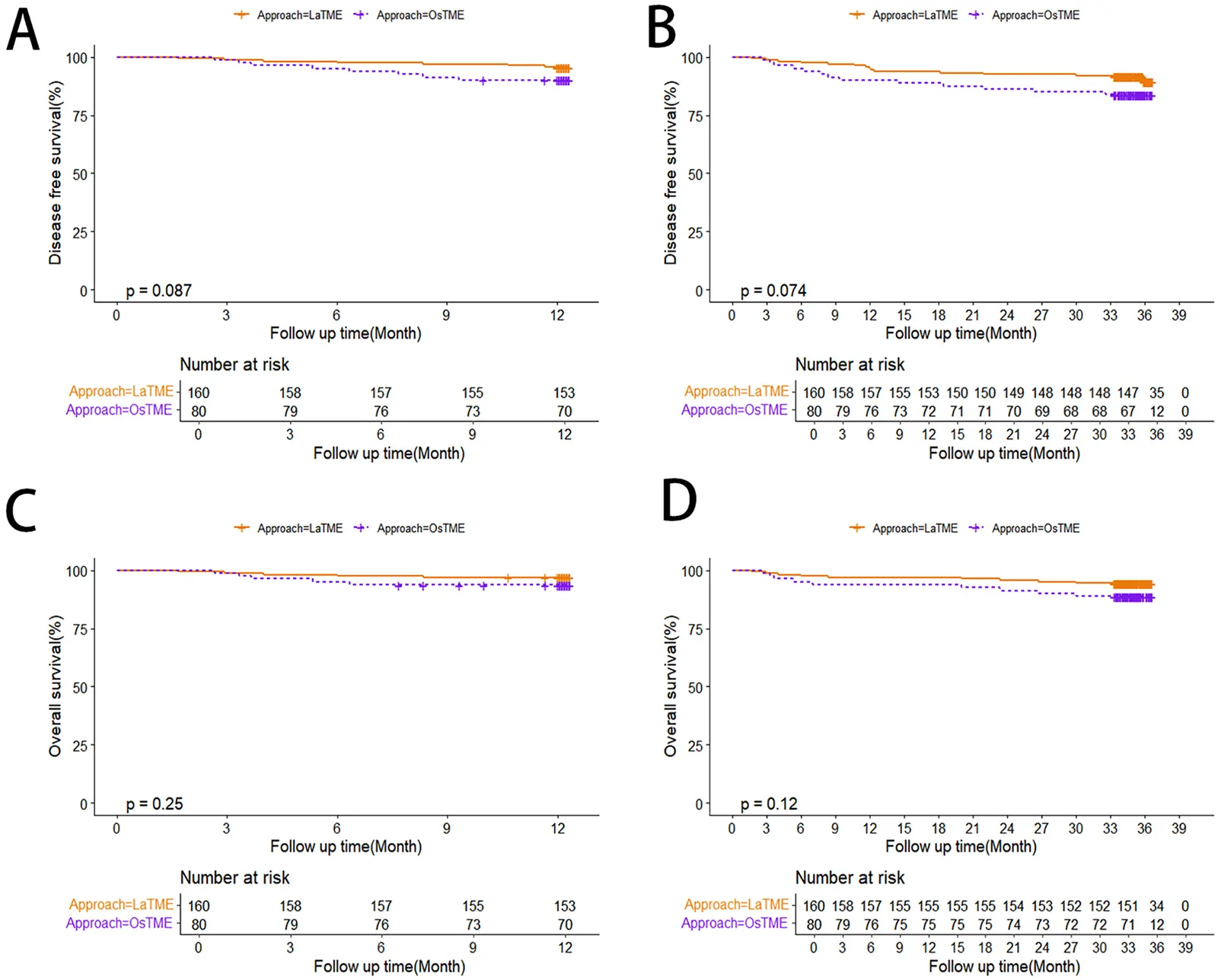
**(A)** The K-M curve of 1-year-DFS after propensity score matching. **(B)** The K-M curve of 3-year-DFS after propensity score matching. **(C)** The K-M curve of 1-year-OS after propensity score matching. **(D)** The K-M curve of 3-year-OS after propensity score matching.

## Discussion

The present study systematically evaluated the short-term clinical efficacies and long-term functional outcomes of LaTME vs. OsTME for mid-low rectal cancer, with a specific focus on surgical procedural details. By utilizing a rigorous propensity score matching methodology, we successfully balanced baseline clinicopathological variables to minimize selection bias, yielding highly comparable cohorts (9). Our analysis revealed that while oncological outcomes and severe systemic complication rates were equivalent between the two surgical modalities, LaTME demonstrated distinct advantages in preserving early postoperative anal function and significantly reducing genitourinary complications, specifically postoperative urinary retention. These findings underscore the profound impact that the meticulous, minimally invasive surgical details afforded by the laparoscopic approach have on postoperative quality of life (10).

A primary concern during the widespread adoption of minimally invasive techniques in colorectal surgery has historically been the potential compromise of oncological radicality (11). However, our data definitively corroborate previous landmark multi-center trials by demonstrating that LaTME is non-inferior to OsTME regarding oncological clearance (12). In our matched cohorts, the achievement of negative circumferential resection margins was impressively high, reaching 97.5% in both the laparoscopic and open groups. Furthermore, macroscopic assessment of surgical specimens confirmed that complete total mesorectal excision was achieved in over 96% of cases regardless of the surgical approach. In terms of long-term oncological efficacy, 1-year and 3-year disease-free survival and overall survival showed no statistically significant differences between the two modalities. This robust oncological equivalence confirms that the minimally invasive approach does not sacrifice curative intent, thereby providing a secure clinical foundation to prioritize functional preservation and enhance the patient's postoperative quality of life.

From the perspective of surgical details, one of the most critical technical advantages of the laparoscopic approach lies in the facilitation of precise vascular dissection, most notably the targeted preservation of the left colic artery (LCA) (13). During the high ligation of the inferior mesenteric artery and the requisite harvesting of the No. 253 lymph nodes at its root, the magnified, high-definition visual field provided by laparoscopy enables surgeons to meticulously skeletonize the vasculature (14). In traditional open surgery, the deep and narrow pelvic cavity, compounded by suboptimal lighting and the obstruction of the surgeon's own hands, often makes isolating the LCA technically demanding and hazardous. Consequently, surgeons may inadvertently sacrifice the vessel to ensure adequate lymphadenectomy and prevent uncontrollable hemorrhage (15). Laparoscopic magnification, acting in concert with advanced energy devices, allows for the clear identification of the LCA origin, facilitating a customized, skeletonized lymph node dissection while safely maintaining the arterial trunk. The preservation of the LCA ensures a robust, pulsatile arterial inflow to the marginal artery of Drummond and the arc of Riolan, thereby maintaining optimal perfusion to the proximal colonic conduit (16). Adequate perfusion is the cornerstone of anastomotic healing. Although our study did not show a statistically significant difference in clinical anastomotic leakage rates, the superior microcirculation afforded by LCA preservation is theoretically pivotal in reducing microscopic ischemia at the anastomotic site. This enhanced local perfusion likely plays a vital role in accelerating the healing of the anastomosis and surrounding pelvic tissues, thereby contributing indirectly to earlier functional recovery and mitigating severe fibrotic strictures that can exacerbate functional bowel syndromes.

Another paramount advantage of LaTME highlighted by our clinical findings is the superior preservation of the pelvic autonomic nerve plexus (PANP), which translates directly into improved postoperative urological outcomes (17). The incidence of postoperative urinary retention in our study was significantly lower in the LaTME cohort compared to the OsTME cohort. The autonomic nerve network—encompassing the superior hypogastric plexus (predominantly sympathetic), the hypogastric nerves, and the pelvic splanchnic nerves (parasympathetic nervi erigentes)—courses precariously close to the fascia propria of the rectum (18). These neural structures govern urinary continence, detrusor contractility, and sexual function. In OsTME, the requirement for excessive blunt dissection and heavy mechanical retraction often causes inadvertent traction injuries, ischemic damage, or direct avulsion of these delicate neural plexuses. Conversely, the laparoscopic approach provides a highly illuminated, magnified, “bottom-up” view of the deep pelvis. The pneumoperitoneum creates natural tension on the areolar tissue planes, facilitating sharp, precise dissection strictly along the “holy plane” of embryological development without breaching the visceral fascia (19). This meticulous precision minimizes both direct mechanical trauma and the lateral spread of thermal energy to the adjacent sympathetic and parasympathetic nerve fibers. Consequently, detrusor motor function and internal sphincter coordination are remarkably better maintained, culminating in the significantly reduced rates of urinary retention observed in our laparoscopic group.

Arguably the most debilitating functional consequence of sphincter-preserving surgery for mid-low rectal cancer is Low Anterior Resection Syndrome (LARS), which severely impacts patients' social functioning and psychological well-being. Our study placed a specific emphasis on this critical metric, utilizing the validated LARS scoring system for systemic, objective quantitative evaluation. We observed a highly statistically significant advantage for LaTME in non-ileostomy patients at one month postoperatively. Specifically, the LaTME cohort experienced a markedly lower incidence of severe LARS compared to the OsTME cohort, with the vast majority of LaTME patients reporting only mild symptoms.

This profound early functional superiority is inextricably linked to the precise surgical execution inherent to laparoscopy in preserving the anal sphincter complex. The preservation of the internal anal sphincter (responsible for the majority of resting anal tone) and the external anal sphincter (critical for voluntary squeeze pressure), along with the levator ani muscle complex, is crucial (20). In open surgery, the confined space of the male or narrow female pelvis often necessitates forceful, prolonged downward traction on the rectum to achieve adequate distal transection margins. This heavy manipulation can inadvertently stretch and tear the pudendal nerve branches and stretch the muscular fibers of the sphincter complex beyond their physiological limits. Furthermore, blind dissection deeper in the pelvis during OsTME increases the risk of damage to the anococcygeal ligament and vital anatomical attachments. Laparoscopic instruments, functioning as slender extensions of the surgeon's hands, offer an unparalleled angle of approach deep down to the levator ani level. The ultra-high-definition camera allows for clear macroscopic distinction between the mesorectal envelope and the pelvic floor musculature, enabling a precise, right-angle transection of the rectum that strictly avoids thermal or mechanical damage to the internal anal sphincter (21). Additionally, the enhanced visualization aids in protecting the intricate local neuronal feedback loops responsible for rectoanal inhibitory reflexes and sensory discrimination within the transitional zone. By minimizing surgical trauma, significantly reducing pelvic floor edema, and avoiding aggressive neuromuscular stretching, LaTME protects the structural and physiological integrity of the anal sphincter complex, thereby substantially reducing the severity of early LARS.

Interestingly, our longitudinal data indicated that this early functional disparity between the two surgical groups gradually diminished over time, with LARS scores successfully equalizing by six months postoperatively. This temporal convergence is consistent with the natural pathophysiological history of LARS, which involves a period of gradual neuroplasticity, neo-rectal reservoir adaptation, and compensatory hypertrophy of the remaining intact sphincter musculature. While the human body's robust compensatory mechanisms eventually bridge the functional gap caused by the slightly greater initial trauma of open surgery, the distinct, highly valuable advantage of LaTME lies in drastically improving the patient's quality of life during the critical, most vulnerable early recovery phase. For the subgroup of patients undergoing defunctioning ileostomy, the delay in restoring bowel continuity likely masks these early traumatic differences entirely, which explains the comparable LARS scores observed between the two groups upon subsequent stoma reversal.

Despite the rigorous implementation of propensity score matching to mitigate baseline imbalances, several limitations within our study must be acknowledged. First, the retrospective nature of the analysis inherently carries the risk of unmeasured confounding variables. Second, as a single-center study, the surgical outcomes may be influenced by the institutional learning curve, standardized perioperative care protocols, and the specific expertise of our surgical team, which may potentially limit the broader generalizability of the findings. Furthermore, while the one- and three-year oncological outcomes are highly promising, a more extended follow-up duration of five to ten years is necessary to comprehensively evaluate definitive oncological safety and the lifelong trajectory of functional bowel recovery.

## Conclusion

Our propensity score-matched analysis confirms that laparoscopic total mesorectal excision provides oncological outcomes equivalent to traditional open surgery for mid-low rectal cancer, alongside superior short-term functional benefits. The enhanced visualization and precision of the laparoscopic approach facilitate the crucial preservation of the left colic artery and pelvic autonomic nerves, significantly reducing postoperative urinary retention. Furthermore, minimized mechanical and thermal trauma to the anal sphincter complex substantially decreases the severity of early Low Anterior Resection Syndrome. These compelling advantages support prioritizing LaTME to maximize functional preservation and postoperative quality of life for rectal cancer patients.

## Data Availability

The raw data supporting the conclusions of this article will be made available by the authors, without undue reservation.
